# Supplementary material for: Synergistic Effect of Dietary Supplementation with Sodium Butyrate, β-Glucan and Vitamins on Growth Performance, Cortisol Level, Intestinal Microbiome and Expression of Immune-Related Genes in Juvenile African Catfish (Clarias gariepinus)
Source: Int J Mol Sci. 2024 Apr 24;25(9):4619. doi: 10.3390/ijms25094619 (PMC11083991; doi:10.3390/ijms25094619)
Supplement: Supplementary file 1 [file ijms-25-04619-s001.zip › ijms-2922855-supplementary.pdf]

Table S1: Relative abundance (%) of bacteria at Phylum and Genus level in the gut microbiome of juvenile African catfish (Clarias gariepinus) fed commercial feed in the control group (C) and fed with enriched feed (W1 – W3) in research groups during a feeding experiment.

	Groups			
**Phylum**	**C (%)**	**W1 (%)**	**W2 (%)**	**W3 (%)**
Actinobacteriota	12.56	10.81	5.89	9.75
Bacteroidota	14.73	6.85	5.74	9.10
Cyanobacteria	0.19	0.13	0.13	0.15
Firmicutes	5.85	6.15	5.01	5.67
Fusobacteriota	51.26	61.44	51.35	54.68
Proteobacteria	15.13	14.22	31.76	20.37
Verrucomicrobiota	0.17	0.26	0.09	0.17
Others	0.10	0.15	0.03	0.10
**Genus**	**C (%)**	**W1 (%)**	**W2 (%)**	**W3 (%)**
Microbacteriaceae	8.14	6.46	3.50	5.25
Aurantimicrobium	2.97	3.28	1.80	2.41
Klugiella	0.81	0.73	0.33	0.20
Bacteroides	1.85	1.71	0.40	0.28
Barnesiellaceae; G: uncultured	7.78	3.57	2.77	3.86
Macellibacteroides	4.76	1.44	2.46	2.58
Anaerorhabdus furcosa group	1.42	1.80	1.70	1.22
Christensenellaceae R-7 group	0.49	1.09	0.06	0.23
Peptostreptococcaceae	1.99	2.01	2.00	1.27
Romboutsia	0.99	0.47	0.69	0.49
Cetobacterium	51.27	61.44	51.35	65.21
Rhizobiales Incertae Sedis; G: uncultured	0.72	0.65	0.34	0.35
GKS98 freshwater group	3.54	2.38	1.48	1.43
Polynucleobacter	0.90	1.41	0.51	2.71
Rhodocyclaceae;g: C39	8.32	9.33	29.04	10.02
Plesiomonas	1.11	0.09	0.17	0.10
Others	2.96	2.13	1.40	2.40
